# Xylo-oligosaccharides improve the adverse effects of plant-based proteins on weaned piglet health by maintaining the intestinal barrier and inhibiting harmful bacterial growth

**DOI:** 10.3389/fmicb.2023.1189434

**Published:** 2023-05-25

**Authors:** Qibing Wang, Yang Zhao, Lei Guo, Xiangyuan Ma, Yi Yang, Yong Zhuo, Xuemei Jiang, Lun Hua, Lianqiang Che, Shengyu Xu, Bin Feng, Zhengfeng Fang, Jian Li, Yan Lin, De Wu

**Affiliations:** Animal Disease-Resistance Nutrition, Ministry of Education, Ministry of Agriculture and Rural Affairs, Key Laboratory of Sichuan Province, Animal Nutrition Institute, Sichuan Agricultural University, Chengdu, Sichuan, China

**Keywords:** xylo-oligosaccharide, weaned piglets, plant-based proteins, gut microbiome, intestinal barrier

## Abstract

**Introduction:**

Piglets are more susceptible to weaning stress syndrome when fed high levels of plant-based proteins that contain abundant food antigens and anti-nutritional factors. Xylo-oligosaccharides (XOS) are a potential prebiotic that may improve the tolerance of weaned piglets to plant-based proteins. The aim of this study was to investigate the effects of XOS supplementation in high and low plant-based protein diets on growth performance, gut morphology, short-chain fatty acid (SCFA) production, and gut microbiota of weaned piglets.

**Methods:**

A total of 128 weanling piglets with an average body weight (BW) of 7.63 ± 0.45 kg were randomly allocated to one of the four dietary treatments in a 2 × 2 factorial arrangement, with two levels of plant-based proteins (d 1–14: 68.3 or 81.33%, d 15–28: 81.27 or 100%) and XOS complex (0 or 0.43%) over a 28-day trial.

**Results:**

The growth performance of piglets did not differ significantly among groups (*P* > 0.05). However, the diarrhea index of weaned piglets fed a high plant-based protein diet (HP) was significantly higher than that of those fed a low plant-based protein diet (LP) at days 1–14 and throughout the experimental period (*P* < 0.05). XOS treatment tended to reduce the diarrhea index at days 1–14 (*P* = 0.062) and during the whole experiment period (*P* = 0.083). However, it significantly increased the digestibility of organic matter at days 15–28 (*P* < 0.05). Moreover, dietary XOS supplementation increased ileal mucosa mRNA expression of *occludin* and *ZO-1* (*P* < 0.05). Furthermore, the concentration of butyric acid (BA) in the cecal contents and in the concentrations of BA and valeric acid (VA) in colon contents were significantly elevated in the XOS groups (*P* < 0.05). Additionally, XOS optimized the gut flora by lowering the number of pathogenic bacteria such as *p_Campylobacterota*, thereby stabilizing the gut ecosystem.

**Discussion:**

In conclusion, the HP diet aggravated diarrhea in weaned piglets while the XOS diet alleviated it by improving nutrient digestibility, protecting intestinal morphology, and optimizing the gut flora.

## 1. Introduction

Before weaning, the main food resource of suckling piglets is highly digestible maternal milk. However, owing to their growth and changes in milk nutrients, piglets are soon confronted with the challenge of weaning, which involves strong weaning stress, leading to high rates of piglet diarrhea and mortality (Hassan et al., 2018; He et al., 2022). Such challenges can be attributed to switching from a liquid to a solid diet, resulting in intestinal morphology changes, including shortening of the intestinal villi and deepening of the crypt, which inhibits digestion and nutrient absorption (Tang X. et al., 2022). Unfortunately, high levels of plant-based proteins in solid diets can further exacerbate weaning stress (Heo et al., 2013), since the undeveloped digestive and immune organs of piglets are less likely to tolerate the abundant food antigens and anti-nutritional factors (ANFs) in plant-based proteins, such as trypsin inhibitors, glycinin, and β-conglycinin, that damage the intestinal barrier and increase intestinal permeability, leading to restricted digestion, absorption, and utilization of nutrients (Jiang et al., 2000) and diarrhea (Sun et al., 2008). Wolfe et al. (2018) mentioned that anaphylaxis and diarrhea could be reduced by lowering plant-based protein levels in the diet of weaned piglets. However, animal-based proteins, such as those from milk and meat, have a significant impact on greenhouse gases in addition to their high price and are known to deplete natural resources (Aiking, 2014; Godfray et al., 2018). Hence, it is of great significance to explore methods to reduce feed costs by lowering the exhaustion of animal protein feed resources, besides relieving the weaning stress of piglets.

Xylo-oligosaccharides (XOS), formed by linking 2–7 xylose units through β-1,4 glycosidic bonds, is a functional polymeric sugar with great probiotic potential (Samanta et al., 2015). XOS has high water solubility and thermal stability, good acidic pH resistance (Wei et al., 2018; Chen et al., 2021a), and no toxicity (EFSA Panel on Dietetic Products et al., 2018). Previous studies have demonstrated that XOS improved the growth performance, immune function, and antioxidant activity of weaned piglets (Pang et al., 2021b). In addition, XOS contributed to maintaining the integrity of the intestinal barrier (Tong et al., 2022), antibacterial activity (Pang et al., 2021a), and anti-inflammatory properties (Ding et al., 2018). The research showed that xylo-oligosaccharides of 100 g/t could significantly increase the content of short-chain fatty acids (SCFA) in piglets (Su et al., 2021). Moreover, the intestinal microbiota alterations of weaned piglets indicated that dietary XOS supplementation could improve intestinal health by increasing the gut microbial diversity and altering the relative abundances of different bacterial species (Ding et al., 2021). However, the impact of XOS on the tolerance of weaned piglets to plant-based proteins remains unclear. Based on the existing functions of XOS, we speculate that it could improve the negative effects of plant protein on the health of piglets. Therefore, we investigated the effects of different levels of plant-based protein diets supplemented with XOS on growth performance, nutrient digestibility, gut morphology, SCFA production, and intestinal microbes of weaned piglets.

## 2. Materials and methods

The XOS complex used in this experiment was independently developed by the Institute of Animal Nutrition, Sichuan Agricultural University, Chengdu, China. The XOS complex was prepared from corn cobs by steam explosion pretreatment combined with enzymatic hydrolysis. The main components of the XOS complex are XOS and silica (the carrier). The results of high-performance liquid chromatography showed that the XOS complex contained 12.3% XOS. The experiment was conducted in the Teaching and Research Center of Sichuan Agricultural University. All animal procedures were approved by the Institutional Animal Care and Use Committee of Sichuan Agricultural University (Ethic Approval Code: SICAU20220801).

### 2.1. Experimental design

A 2 × 2 factorial arrangement was employed involving two levels of plant-based protein (plant-based proteins account for 68.3% of the total protein for the LP group and 81.33% of the total protein for the HP group at days 1–14; plant-based proteins account for 81.27% of the total protein for the LP group and 100% of the total protein for the HP group at days 15–28) and two concentrations of XOS complex (0 and 0.43%). The effective content of XOS in the diet was 450 mg/kg (Chen et al., 2021b). A total of 128 piglets [(Landrace × Yorkshire) × Yorkshire, 25 days ± 2days] with an initial body weight of 7.63 ± 0.45 kg were randomly assigned into the four treatment groups in a randomized complete block design according to body weight and gender. There were eight pens in each treatment group, each housing four piglets (two barrows and two gilts) for the 28-day experiment.

### 2.2. Feeding management

As shown in Table 1, experimental diets were prepared according to the nutritional requirements of piglets at body weights of 7–11 kg and 11–25 kg (National Research Council, 2012; USA). The experiment lasted for 28 days and was divided into two stages: days 1–14 (stage 1) and days 15–28 (stage 2). Diets at the same stage were formulated to be iso-nitrogenous and iso-energetic. Cleaning and disinfection of pens were performed before starting the experiment. During the experiment, the room temperature was maintained at ~28°C, and all piglets had free access to feed and water.

**Table 1:** Ingredients composition and nutrient level of the diet (as-fed basis).

**Items**	**Stage 1 (1–14 days)**		**Stage 2 (15–28 days)**	
	**LP**	**HP**	**LP**	**HP**
**Ingredients, %**				
Corn	45.74	43.54	51.62	48.87
Broken rice	20.00	20.00	20.00	20.00
SPC	4.00	4.00	3.90	3.90
Soybean meal	10.25	16.00	13.90	21.02
Fish meal	5.00	2.50	3.00	–
Chicken liver powder	3.00	1.50	2.00	–
Whey powder	5.00	5.00	–	–
Soybean oil	2.39	2.48	1.32	1.50
Sucrose	2.00	2.00	2.00	2.00
Limestone	0.71	0.71	0.68	0.65
CaHPO_4_	0.46	0.78	0.46	0.86
NaCl	0.20	0.20	0.20	0.20
L-Lysine·HCl, 98.5%	0.39	0.41	0.32	0.36
DL-Methionine, 98.5%	0.06	0.08	0.05	0.08
L-Threonine, 98.5%	0.04	0.05	0.03	0.05
L-Tryptophan, 98.5%	0.06	0.05	0.02	0.01
Choline chloride	0.15	0.15	0.15	0.15
Vitamin premix^a^	0.05	0.05	0.05	0.05
Mineral premix^b^	0.30	0.30	0.30	0.30
ZnO, 65%	0.20	0.20	-	-
**Nutrient composition** ^c^				
DE, cal/g	3540.00	3540.00	3490.00	3490.00
CP, %	18.98	18.98	18.50	18.50
Ca, %	0.80	0.80	0.70	0.70
Total P, %	0.59	0.60	0.53	0.55
Lysine,%	1.35	1.35	1.23	1.23
Methionine, %	0.39	0.39	0.36	0.36
Threonine, %	0.79	0.79	0.74	0.74
Tryptophan, %	0.26	0.26	0.22	0.22

SPC, Soybean concentrate protein; DE, Digestible energy; CP, Crude protein.

^a^Vitamin premixes provided per kilogram of diet: vitamin A, 15,000 IU; vitamin D_3_, 5,000 IU; vitamin E, 40 mg; vitamin K_3_ 5 mg; vitamin B_1_ 5 mg; vitamin B_2_ 12.5 mg; vitamin B_6_ 6 mg; vitamin B_12_ 0.06 mg; nicotinic acid, 50 mg; pantothenic acid, 25 mg; folic acid, 2.5 mg; biotin, 0.25 mg.

^b^Mineral premix provided per kilogram of diet: Cu 6 mg as CuSO_4_·5H_2_O; Fe 100 mg as FeSO_4_·7H_2_O; I 0.14 mg as KI; Zn 100 mg as ZnSO_4_·7H_2_O; Mn 20 mg as MnSO_4_·H_2_O; Se 0.3 mg as Na_2_SeO_3_·5H_2_O.

^c^Nutrient levels are calculated values.

### 2.3. Sample collection

After 28 days of the experiment, we chose one piglet with an average weight from each pen and slaughtered it. The intestinal tissues were removed and separated into the duodenum, jejunum, and ileum. A piece of ~3 cm from the middle of each intestinal segment was immediately excised and stored in a 4% paraformaldehyde solution for the determination of the intestinal tissue morphology. The ileum mucosa and the contents of the ileum and colon were collected and packed with cryogenic tubes. Samples were immediately frozen in liquid nitrogen and stored at −80°C.

To determine nutrient digestibility in the two stages, 0.3% chromium trioxide was added to diets at weeks 2 and 4. The first 4 days of both weeks 2 and 4 were defined as the adaptation period, and fresh fecal samples were collected and immediately stored at −20°C during the last 3 days.

### 2.4. Growth performance

The BW of individual piglets was measured at the start and at the end of each stage of the experiment, and the feed consumed and rejected by each piglet was recorded daily to calculate performance parameters including average daily feed intake (ADFI), average daily gain (ADG), and feed to gain ratio (F:G) of piglets at each stage and for the whole period.

Diarrhea was scored visually at 8:00, 12:00, and 16:00 h each day during the trial. In brief, firm and well-formed feces were scored as 0; soft but formed feces were scored as 1; sticky and liquid feces were scored as 2; watery and projectile feces were scored as 3. The diarrhea index was calculated using the formula: diarrhea index = sum of diarrhea scores/(number of piglets per pen × experimental days × assessed times per day).

### 2.5. Chemical analysis and calculation

Samples of diets and feces were freeze-dried in a freeze-drying machine for further determination of gross energy (GE), dry matter (DM), organic matter (OM), and crude protein (CP). The GE was measured by an automatic adiabatic oxygen bomb calorimeter (Parr 64001101-22141, Parr Instrument Co., Moline, IL, 61265USA). Ash and DM were analyzed based on the AOAC method (AOAC, 2007; Gaithersburg). CP was determined by the copper catalyst Kjeldahl method. Chromium was determined using a ContrAA 700 fire flame atomic absorption spectrophotometer (Analytik Jena GmbH, Jena, Germany). Apparent total tract digestibility (ATTD) was calculated according to the following equation: ATTD_nutrient_ = 1 – (Cr_diet_ × Nutrient_feces_)/(Cr_feces_ × Nutrient_diet_) (Li Y. et al., 2021).

### 2.6. Gene expression

Total RNA from ileal mucosa was extracted using RNAiso Plus TRIzol reagent (Takara Bio Inc., Japan). The quality and concentration of extracted total RNA were then determined by 1% agarose gel electrophoresis and ultraviolet spectrophotometry using a NanoDrop 2000 instrument (Thermo Fisher Scientific, USA). A HiScript III RT SuperMix for qPCR kit (Vazyme Code: R323-01) was used to remove genomic DNA. cDNA was obtained by reverse transcription using a PCR instrument (NanoDrop 2000, Thermo, United States). Finally, quantitative RT-PCR tests were performed using ChamQ SYBR Color qPCR Master Mix (Vazyme Code: Q43102) and the ABI7900HT reaction program (Vazyme, Q711, Nanjing, China). With GAPDH as the internal reference gene, the 2^−Δ*ΔCt*^ method was used to analyze real-time qPCR data. Target gene primer sequences are shown in Supplementary Table S1.

### 2.7. Small intestinal morphology

Morphological samples of the duodenum, jejunum, and ileum were sent to Yibaidao Technology Co., Ltd. (Chengdu, China) for further analysis. In brief, samples were embedded in paraffin and stained, and at least two 100-fold fields were randomly selected to be photographed for each section of each treatment group. Afterward, Image-Pro Plus 6.0 software (Media Cybernetics, Inc., Rockville, MD, USA) was used to analyze villus height (VH) and crypt depth (CD).

### 2.8. Quantification of SCFAs

The concentrations of acetic acid (AA), propionic acid (PA), butyric acid (BA), and valerate acid (VA) in the cecum and the colon were determined by gas chromatography (Varian CP-3800, manual injection, flame ionization detector, FID, 10 μL micro-injector). Briefly, 0.5 g samples and 1.2 mL of ultra-pure water were placed in a centrifuge tube, shaken, and mixed using a cryogenic tissue grinder (XOYM 24DL). After keeping the tubes for 30 min at room temperature, they were centrifuged at 1,000 × g for 15 min before 1 mL of supernatant was removed and mixed with 0.2 mL 25% metaphosphate and 23.3 μL 210 mM crotonate. After storing the samples for another 30 min at room temperature, they were centrifuged at 8,000 × g for 10 min, and 0.2 mL of the supernatant was removed and mixed with 0.6 mL of a methanol solution. Lastly, after centrifuging at 8,000 × g for 5 min, the supernatant was passed through a 0.22 μm filter membrane (Millipore Co., Bedford, MA).

### 2.9. Sequencing of gut microbiome

Colonic chyme was sent to Novogene Technology Co., Ltd. (Beijing, China) for 16S RNA sequencing. Briefly, genomic DNA was extracted, and the purity and concentration were determined. PCR was then carried out to generate amplification products that were purified before library construction and detection. The resulting data were spliced, filtered, and denoised to generate clean data. We conducted operational taxonomic unit (OTU) clustering on clean data and determined the community composition of each sample at different classification levels. We also used QIIME software (Version 1.9.1) to calculate observed_species, Chao1, Simpson, abundance-based coverage estimator (ACE) indices, and UniFrac distance. Principal co-ordinate analysis (PCoA) was then performed based on the unweighted UniFrac distance. The linear discriminant analysis (LDA) effect size (LEfSe) method was used to analyze differences between treatments. Correlations between differential microbiota (at the phylum and genus levels) and diarrhea index or SCFA contents were evaluated by Spearman's correlation test using the R language package “Pheatmap”.

### 2.10. Statistical analysis

Data were analyzed using the MIXED procedure of SAS 9.4 (SAS Institute Inc., Cary, NC, USA). Before the parameter analysis, the normality and homogeneity of the residuals should be tested. A 2 × 2 factorial arrangement was employed for pens as experimental units. The model included the effects of dietary and XOS treatments and their interactions. When there were major effects and interaction effects, the LSD method was used to compare the significance between the means of each group. Different microbial taxa were identified using LEfSe with LDA score of >3.5. All data presented in the table are expressed as mean and ensemble standard error (SEM). The correlation analysis was performed by Spearman's correlation test (Best and Roberts, 1975). The results were considered significant at a *P*-value of < 0.05, and the tendency toward significance was considered at 0.05 ≤ *P* < 0.10.

## 3. Results

### 3.1. Growth performance

Compared with weaned piglets fed an LP diet, weaned piglets fed an HP diet had significantly higher diarrhea indices at days 1–14 and 1–28 (*P* < 0.05). XOS consumption tended to decrease the diarrhea index at days 1–14 (*P* = 0.062) and 1–28 (*P* = 0.083), and there was an interaction effect between dietary treatment and XOS treatment on the diarrhea index at days 15–28 (*P* = 0.056). However, neither dietary treatment nor XOS treatment had significant effects on ADFI, ADG, or F/G of weaned piglets (*P* > 0.05, Table 2).

**Table 2:** Effects of different levels of plant-based proteins diets supplemented with XOS on the growth performance and diarrhea index in weaned piglets.

**Item**	**LP**		**HP**		**SEM**	***P*** **-value**		
	**XOS-**	**XOS**+	**XOS-**	**XOS**+		**Diet**	**XOS**	**Diet** × **XOS**
**BW, kg**								
Day 1	7.64	7.62	7.61	7.63	0.45	0.976	0.998	0.972
Day 14	9.67	9.85	9.73	9.80	0.60	0.999	0.838	0.929
Day 28	12.80	12.93	12.95	12.88	0.72	0.953	0.969	0.891
**Days 1–14**								
ADFI, g/d	292.58	306.51	311.13	305.75	23.43	0.707	0.856	0.684
ADG, g/d	145.27	159.21	151.35	155.20	15.88	0.948	0.580	0.753
F:G	1.96	2.10	2.16	2.02	0.13	0.654	0.321	0.993
Diarrhea index	0.40^b^	0.31^b^	0.59^a^	0.48^ab^	0.05	0.002	0.062	0.812
**Days 15–28**								
ADFI, g/d	517.37	535.91	523.58	549.21	38.59	0.802	0.572	0.927
ADG, g/d	240.89	237.02	247.64	236.74	16.95	0.850	0.667	0.837
F:G	2.15	2.29	2.15	2.36	0.13	0.789	0.179	0.808
Diarrhea index	0.28	0.34	0.66	0.32	0.10	0.075	0.169	0.056
**Days 1–28**								
ADFI, g/d	404.98	421.21	417.35	427.48	29.08	0.650	0.760	0.807
ADG, g/d	193.06	198.12	199.46	195.97	13.92	0.881	0.938	0.759
F:G	2.12	2.13	2.15	2.19	0.09	0.372	0.801	0.905
Diarrhea index	0.35^b^	0.32^b^	0.63^a^	0.41^ab^	0.06	0.010	0.083	0.147

BW, Body weight; ADFI, average daily feed intake; ADG, average daily gain; F:G, feed-to-gain ratio.

^a, b^Mean values within a row with different letters differ at a *P*-value of < 0.05.

### 3.2. ATTD of nutrients

Compared with the LP diet, with an HP diet, the digestibility of CP tended to decrease (*P* = 0.061) during the first stage, and during the second stage, the digestibility of GE (*P* = 0.066) and OM (*P* = 0.058) tended to decrease but the digestibility of CP was significantly decreased (*P* < 0.05). Dietary addition of XOS tended to increase DM digestibility (*P* = 0.059) and GE digestibility (*P* = 0.062), and it significantly increased OM digestibility of weaned piglets during the second stage (*P* < 0.05). In addition, the interaction effects between dietary treatment and XOS treatment on DM digestibility, OM digestibility, and GE digestibility of weaned piglets were evident during the first stage (*P* < 0.05, Table 3).

**Table 3:** Effects of different levels of plant-based proteins diets supplemented with XOS on ATTD of nutrients in weaned piglets.

**Item**	**LP**		**HP**		**SEM**	***P*** **-value**		
	**XOS-**	**XOS**+	**XOS-**	**XOS**+		**Diet**	**XOS**	**Diet** × **XOS**
**Stage 1**								
DM, %	84.24	82.31	82.24	82.59	0.58	0.152	0.184	0.059
OM, %	88.24^a^	86.64^b^	86.64^b^	87.12^ab^	0.46	0.238	0.239	0.034
CP, %	74.93	71.88	71.50	71.16	1.06	0.061	0.122	0.210
DE, %	83.51	81.74	81.35	82.11	0.73	0.229	0.492	0.095
**Stage 2**								
DM, %	85.43	86.43	84.81	86.73	0.73	0.829	0.059	0.533
OM, %	89.13^b^	89.97^a^	88.81^b^	89.24^ab^	0.26	0.058	0.026	0.437
CP, %	78.15	78.66	77.32	76.68	0.67	0.049	0.922	0.399
DE, %	85.38	86.23	84.67	85.39	0.40	0.066	0.062	0.869

DM, Dry matter; CP, Crude protein; DE, Digestible energy; OM, organic matter; Stage 1, days 1–14; Stage 2, days 15–28.

^a, b^Mean values within a row with different letters differ at a P-value < 0.05.

### 3.3. Intestinal morphology

No significant interaction was observed between dietary treatment and XOS treatment on VH, CD, and VH:CD in the duodenum and jejunum of piglets (*P* > 0.05). However, VH tended to increase in the ileum of piglets fed aXOS diet (*P* = 0.075, Table 4; Supplementary Figure S1).

**Table 4:** Effects of different levels of plant-based proteins diets supplemented with XOS on the intestinal morphology in weaned piglets.

**Item**	**LP**		**HP**		**SEM**	***P*** **-Value**		
	**XOS-**	**XOS**+	**XOS-**	**XOS**+		**Diet**	**XOS**	**Diet** × **XOS**
**Duodenum**								
VH, μm	569.53	603.62	595.25	626.57	29.61	0.419	0.280	0.963
CD, μm	120.47	127.06	126.11	140.31	9.46	0.328	0.283	0.691
VH:CD	4.86	4.82	4.86	4.58	0.34	0.719	0.650	0.736
**Jejunum**								
VH, μm	529.53	504.87	510.08	507.31	25.10	0.738	0.589	0.667
CD, μm	126.22	136.75	124.84	129.15	7.04	0.529	0.302	0.663
VH:CD	4.35	3.77	4.09	4.02	0.32	0.980	0.322	0.437
**Ileum**								
VH, μm	324.28	353.84	330.41	417.50	31.36	0.277	0.075	0.368
CD, μm	102.18	115.44	105.24	118.28	8.81	0.741	0.149	0.990
VH:CD	3.22	3.10	3.24	3.58	0.25	0.329	0.673	0.383

VH, Villous height; CD, Crypt depth.

### 3.4. Ileal expression of genes related to barrier functions

**Figure 1:**
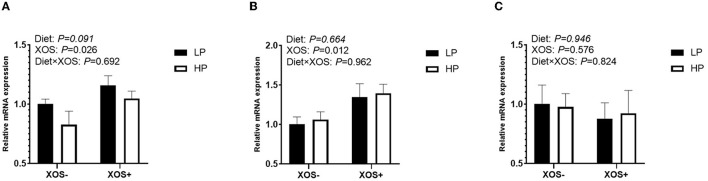
The relative mRNA expression of intestinal epithelium integrity-related genes in the ileal tissues of piglets. **(A)** *ZO-1*, **(B)** *Occludin*, and **(C)** *claudin-1*.

The function of tight junctions of the intestinal barrier was tested by analyzing the gene expression of ileal *occludin, claudin-1*, and *ZO-1* (Figures 1A–C). Although the HP diet tended to decrease the expression of *ZO-1* compared with the LP diet in the ileum of piglets (*P* = 0.091), no significant effect of diet on the expression levels of *occludin* and *claudin-1* in the ileum was observed. In addition, XOS significantly increased the relative expression levels of *occludin* and *ZO-1* (*P* < 0.05) but had no significant effect on *claudin-1*.

### 3.5. SCFA levels

Compared with the HP diet, the LP diet significantly increased the concentration of VA in colonic contents (*P* < 0.05). On the one hand, XOS treatment tended to increase AA concentration in the cecal contents of piglets (*P* = 0.075). On the other hand, the levels of BA in cecal contents and those of BA and VA in colonic contents in the XOS+ treatment groups were significantly higher than in those in the XOS- treatment groups regardless of the dietary treatment (*P* < 0.05, Table 5).

**Table 5:** Effects of different levels of plant-based proteins diets supplemented with XOS on SCFAs in weaned piglets.

**Item**	**LP**		**HP**		**SEM**	***P*** **-Value**		
	**XOS-**	**XOS**+	**XOS-**	**XOS**+		**Diet**	**XOS**	**Diet** × **XOS**
**Cecal chyme**								
AA, μmol/mg	3.56	3.84	3.44	4.14	0.26	0.737	0.075	0.449
PA, μmol/mg	1.73	1.92	1.67	1.85	0.11	0.532	0.107	0.967
BA, μmol/mg	0.86^c^	1.18^a^	0.87^bc^	1.10^ab^	0.09	0.719	0.004	0.590
VA, μmol/mg	0.23	0.33	0.26	0.26	0.04	0.570	0.280	0.220
**Colon chyme**								
AA, μmol/mg	3.44	3.88	3.54	3.76	0.24	0.970	0.183	0.656
PA, μmol/mg	1.74	1.69	1.49	1.72	0.16	0.495	0.587	0.400
BA, μmol/mg	1.08^ab^	1.44^a^	0.72^b^	1.00^b^	0.14	0.009	0.034	0.759
VA, μmol/mg	0.34	0.47	0.26	0.36	0.05	0.107	0.043	0.752

^a−*c*^Means in a row with different superscripts that differ significantly (*P* < 0.05). AA, acetate acid; PA, propionate acid; BA, butyric acid; VA, valerate acid.

### 3.6. Changes in gut microbiome diversity

**Figure 2:**
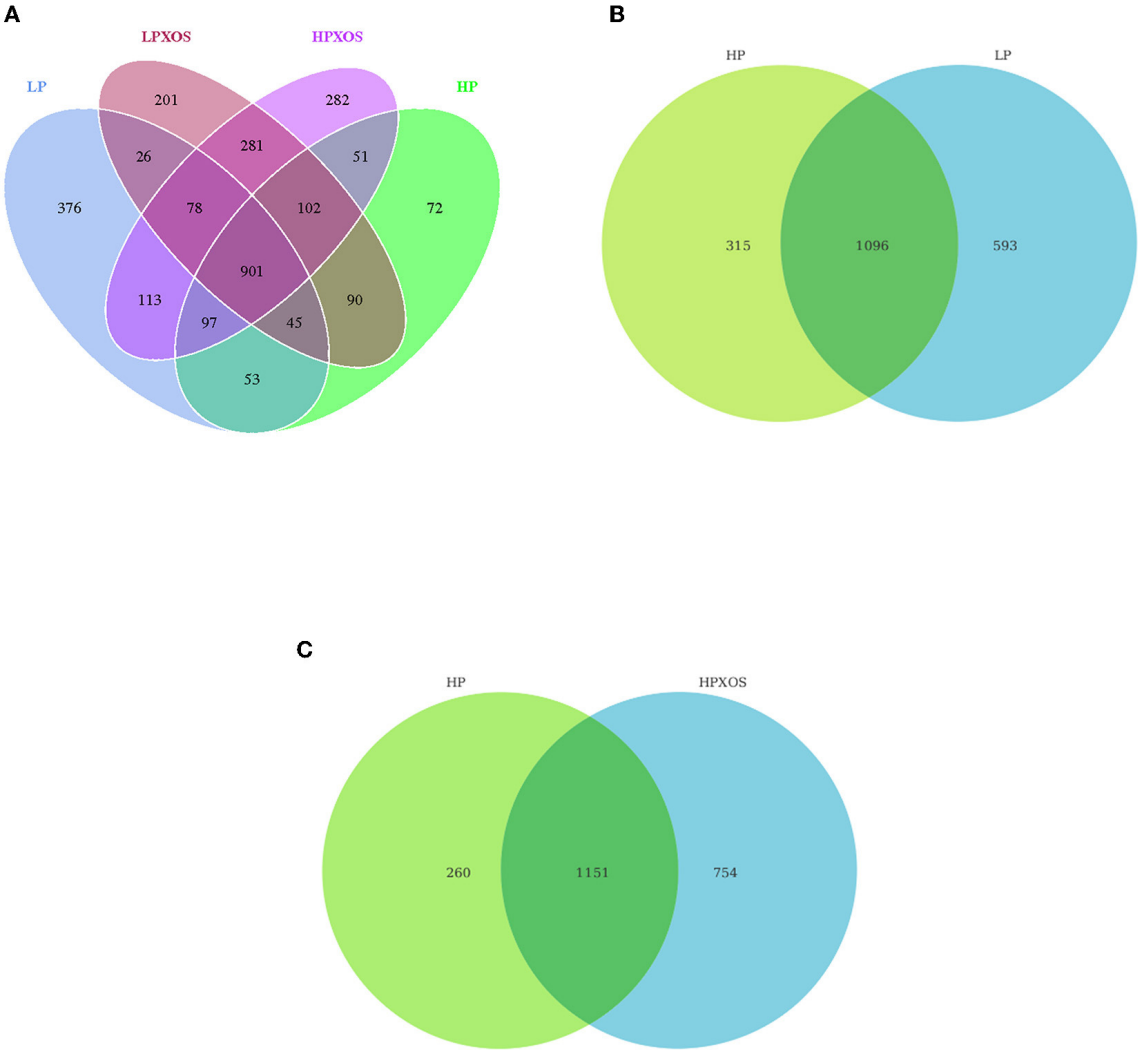
Microbiota comparison of the OTUs among treatments in the colon. The observed OTUs share ≥ 97% sequence similarity. **(A–C)** Venn diagrams showing the unique and shared OTUs in the different groups. LP, low plant-based proteins, no XOS; HP, high plant-based proteins, no XOS; LPXOS, low plant-based proteins with XOS; HPXOS, high plant-based proteins with XOS.

According to the OTU results obtained by clustering, we analyzed common and unique OTUs among different treatment groups and visualized the distribution of OTUs among different treatment groups using a Venn diagram. As shown in Figure 2A, 1,689, 1,411, 1,724, and 1,905 OTUs were, respectively, observed in the LP, HP, LP + XOS, and HP + XOS groups, and there were 901 identical OTUs in all four treatment groups. It is evident that the number of OTUs of piglets treated with the LP diet was higher than those of piglets treated with the HP diet (Figure 2B), and OTUs of the HP + XOS group were more abundant than those of the HP group (Figure 2C). However, there was no interaction effect on the number of observed species in each group.

**Figure 3:**
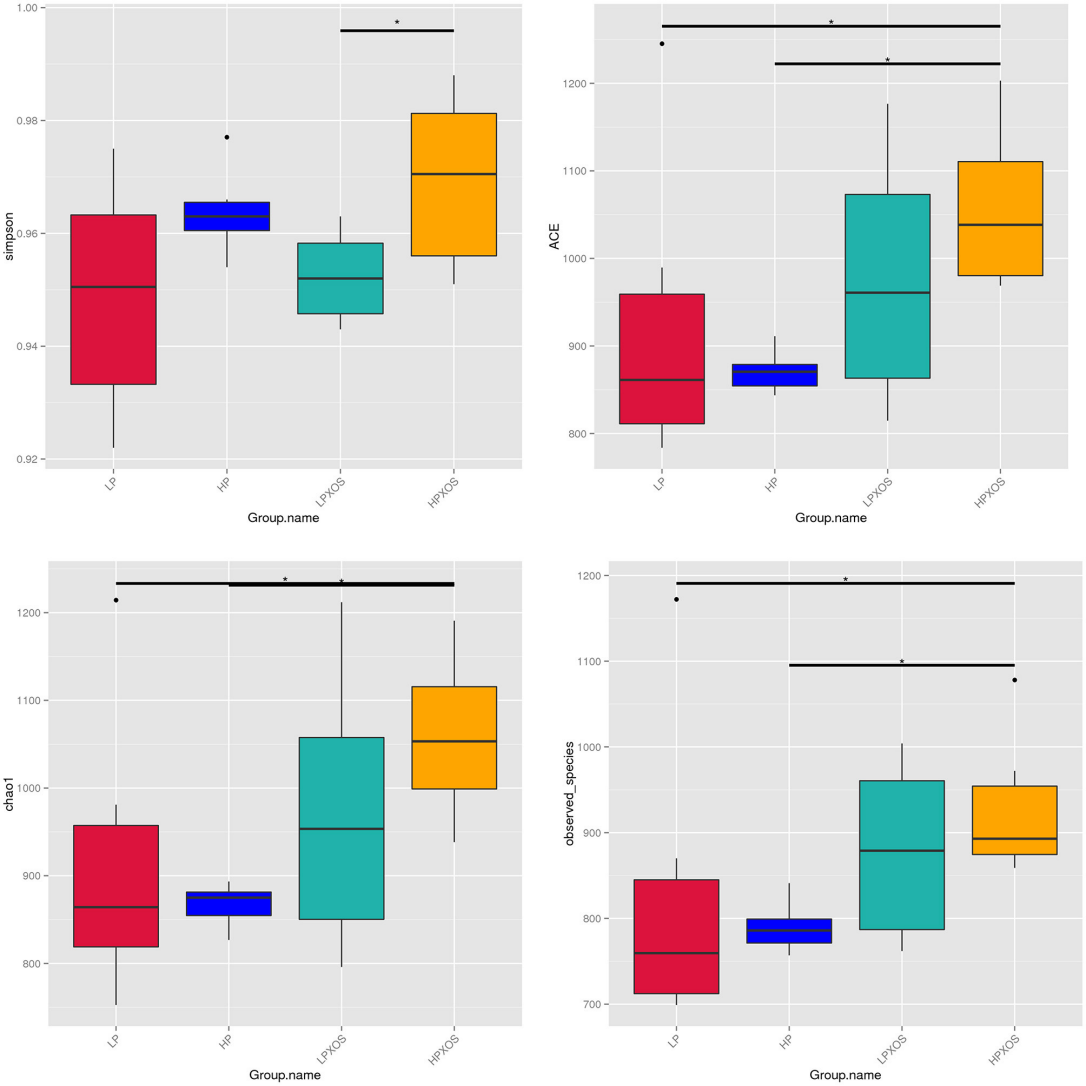
Microbiota alpha-diversity comparison among four groups. Piglets were regarded as the experimental units, *means at a *P*-value of <0.05. LP, low plant-based proteins, no XOS; HP, high plant-based proteins, no XOS; LPXOS, low plant-based proteins with XOS; HPXOS, high plant-based proteins with XOS.

**Figure 4:**
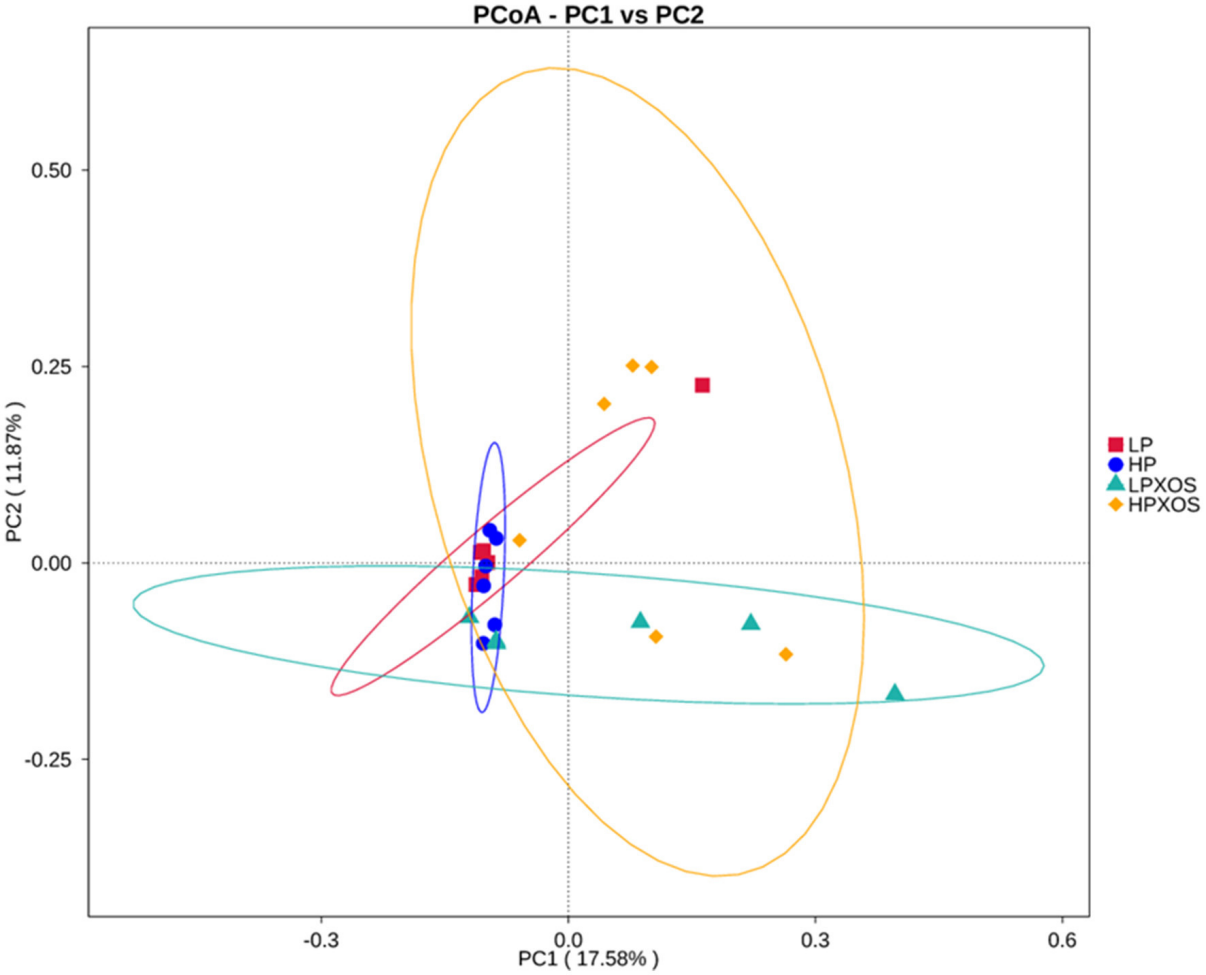
Comparison of the gut microbiota composition among four groups. Unweighted UniFrac Principal Co-ordinates Analysis (PCoA) based on OTUs of the gut microbiome. LP, low plant-based proteins, no XOS; HP, high plant-based proteins, no XOS; LPXOS, low plant-based proteins with XOS; HPXOS, high plant-based proteins with XOS.

To probe the richness and diversity of the microbial communities of groups, alpha-diversity indices (observed_species, Chao1, Simpson, and ACE) were analyzed (Figure 3). The XOS treatment significantly increased Simpson, Chao1, and ACE indices (*P* < 0.05), and it tended to increase observed_species (*P* = 0.056). However, dietary treatment had no significant effect on the alpha-diversity index (*P* > 0.05). No interaction between the dietary and XOS treatments was observed for the microbial parameters described above. PCoA revealed that the colonic microbial communities of different dietary and XOS treatments were separately aggregated and had significantly different community structures (Figure 4).

### 3.7. Relative abundance of colonic chyme microorganisms and the correlations between bacterial abundance and diarrhea index or SCFA contents

**Figure 5:**
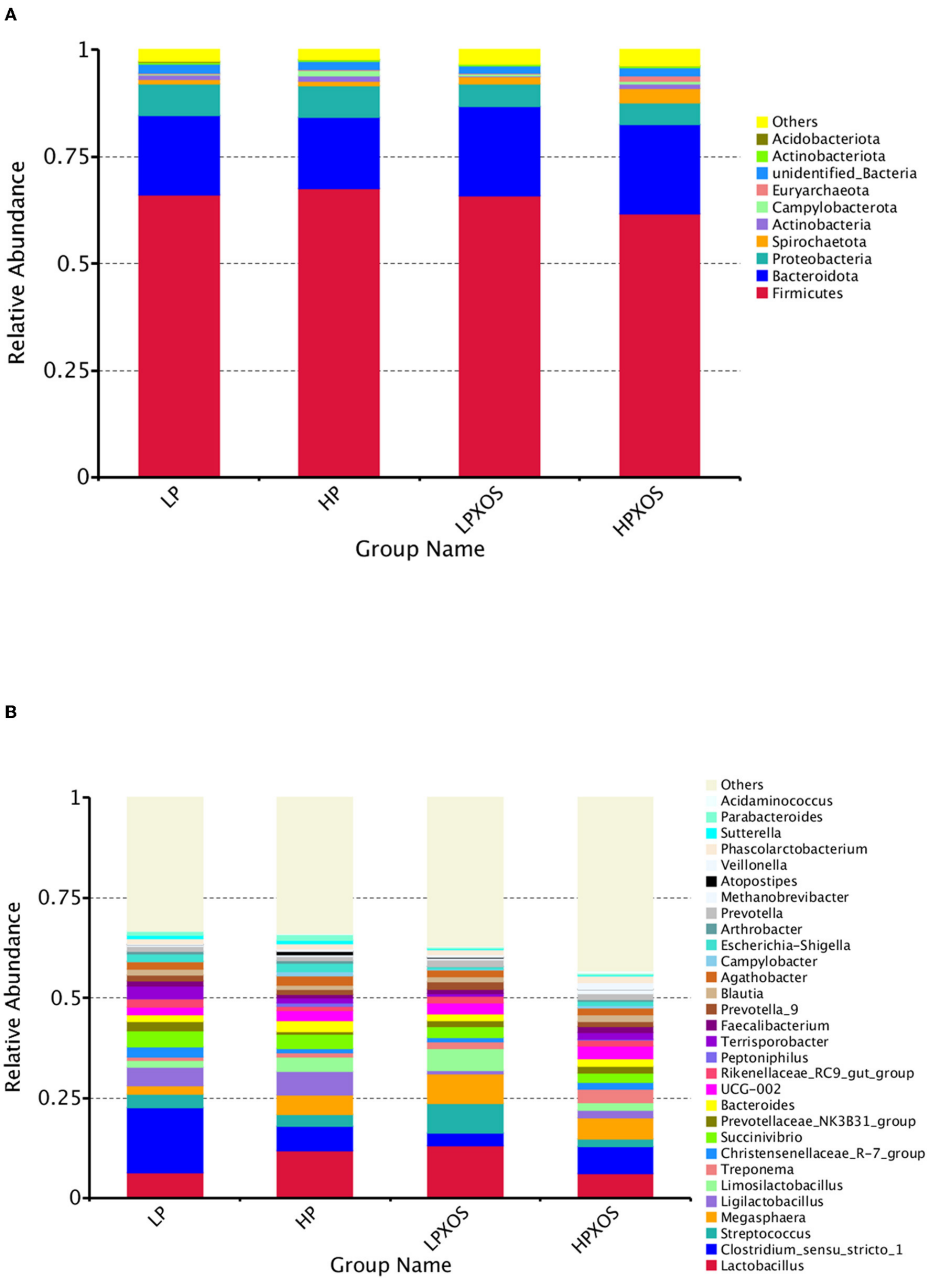
Effects of different levels of plant-based proteins diets supplemented with XOS on the phyla and genera of the gut microbiome in weaned piglets. Relative abundances of phyla **(A)** and genus **(B)**. The abundance is expressed in terms of a percentage of the total effective bacterial sequences in the fecal samples.

According to the species annotation results, the top ten colonic flora species with maximum abundance in each group at the phylum level were selected to generate a columnar accumulation diagram of relative species abundance (Figure 5A). Compared with piglets fed the LP diet, the relative abundance of *Campylobacterota* was increased significantly in piglets fed the HP diet (*P* < 0.05). The XOS treatment tended to decrease *Proteobacteria* (*P* = 0.097) and increase *Euryarchaeota* (*P* = 0.093). However, no significant interaction was observed between dietary and XOS treatments (*P* > 0.05).

At the genus level, the top 30 colonic flora species with maximum abundance in each group were selected to generate a columnar accumulation diagram of relative abundance (Figure 5B). Compared with piglets fed the LP diet, the relative abundance of *Campylobacter* was increased significantly in piglets fed the HP diet (*P* < 0.05). The addition of XOS significantly reduced (*P* < 0.05) the relative abundance of *Clostridium_sensu_stricto_1, Ligilactobacillus, Terrisporobacter*, and *Escherichia-Shigellain* in the colon contents of piglets.

**Figure 6:**
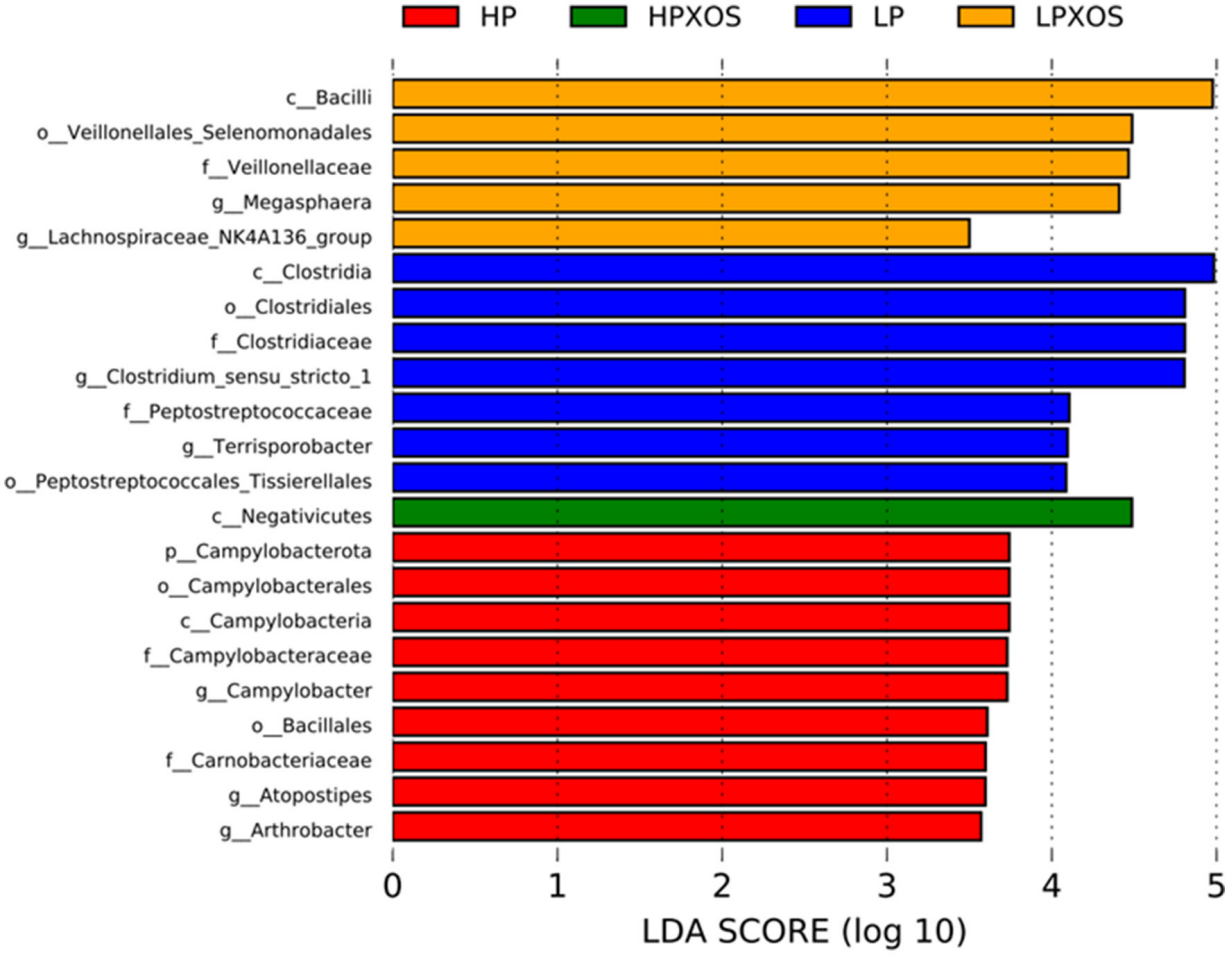
Effects of different levels of plant-based protein diets supplemented with XOS on the distribution of the phylotypes in weaned piglets. LefSe bar representing differential abundant taxa in pig gut microbiome.

Species with significant abundance differences in different treatment groups were analyzed by LEfSe. A total of 7, 9, 5, and 1 dominant bacteria were observed in colonic chyme samples of the LP, HP, LP+XOS, and HP+XOS groups, respectively (Figure 6). Note that pathogenic bacteria were more dominant in the HP group than in the HP + XOS group.

**Figure 7:**
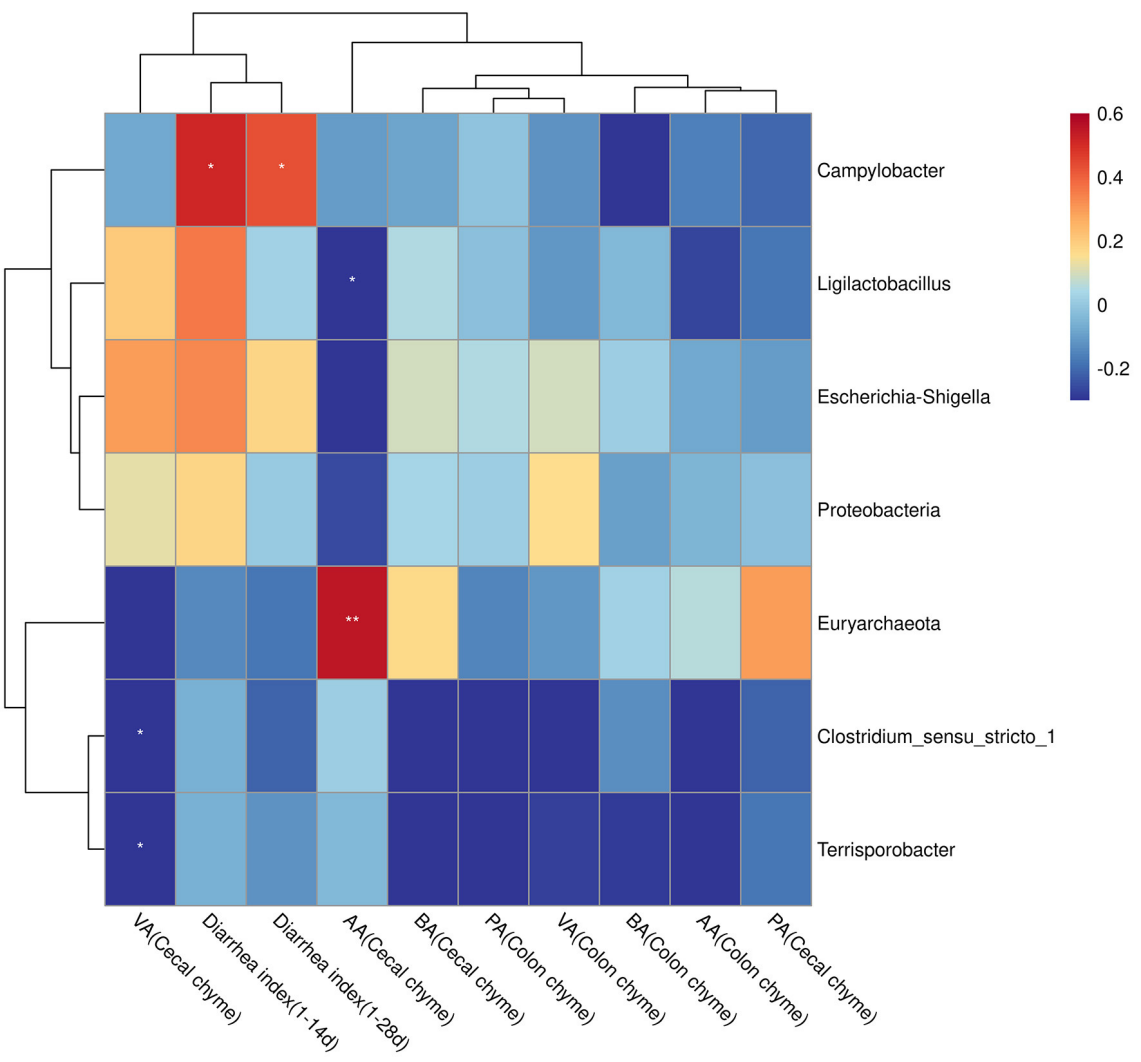
Correlations between bacterial abundance and diarrhea index or SCFA contents. The red color represents a positive correlation, and the blue color represents a negative correlation. *P < 0.05; **P < 0.01; AA, acetate acid; PA, propionate acid; BA, butyric acid; VA, valerate acid.

Diarrhea index (at days 1–14 and 1–28) positively correlated with *Campylobacter* (*P* < 0.05). The valerate content in the cecal chyme was negatively correlated with *Clostridium_sensu_stricto_1* and *Terrisporobacter* (*P* < 0.05). Acetate content in the cecal chyme was negatively correlated with *Ligilactobacillus* (*P* < 0.05) and positively correlated with *Euryarchaeota* (*P* < 0.01, Figure 7).

## 4. Discussion

Protein is one of the most important nutrients for life and a key factor determining whether a diet is healthy or not (Wolfe et al., 2018). The selection of dietary protein sources for weaned piglets, especially the application of plant-based proteins, is important in the pig industry because high levels of plant-based proteins in the diet can cause intestinal damage in piglets due to the influence of antigen proteins; this affects the digestion and utilization of protein, the incidence of diarrhea, and the growth performance, resulting in economic losses to animal husbandry (Xia et al., 2022). In our experiment, the dietary treatment had no significant effect on the growth performance of weaned piglets. Similarly, Li Y. et al. (2021) found no significant difference in the growth performance of piglets between different treatment groups when replacing fish meal and milk powder with enzymatically hydrolyzed soy. In contrast, another study showed that replacing animal protein with soybean protein concentrate could significantly reduce BW, ADG, and ADFI of piglets (Deng et al., 2022). The inconsistency of test results may be caused by differences in substitution ratio and substituted raw materials. It has been suggested that dietary protein digestion is a major factor affecting the intestinal development and diarrhea rate of weaner piglets (Li et al., 2019), since undigested protein can enter the large intestine and disrupt intestinal microorganisms (Zhao et al., 2019). In our experiment, although there was no difference in growth performance among treatment groups, the diarrhea index of piglets treated with HP diet was higher than that of those treated with LP diet, indicating adverse effects of high plant protein levels on weaned piglets, which may be due to the lower CP digestibility of HP diet than LP diet.

The role of XOS as a prebiotic is well known, but research on different plant-based protein levels is lacking. In our experiment, XOS supplementation at different levels of plant protein had no significant effect on the growth performance of weaned piglets. Previous studies have reported inconsistent conclusions regarding the role of XOS in the growth performance of weaned piglets. For example, adding 500 mg/kg XOS with 95% purity to the diet of weaned piglets significantly increased BW, ADG, ADFI, and feed conversion efficiency (Chen et al., 2021b). However, Yin et al. (2019) found that dietary supplementation of 0.01% XOS with 40% purity had no effect on the growth performance of piglets, consistent with our current results. However, the diarrhea index of the HP group was significantly increased throughout the experiment period compared with the LP group, but there was no significant difference between HP + XOS and LP groups. This demonstrates the capacity of XOS to ameliorate the adverse effects of plant protein on piglets to a certain extent.

We found that dietary XOS effectively improved ATTD. Previous studies have reported that dietary supplementation of XOS (200 mg/kg) increased ATTD of DM, nitrogen, and GE in weaned piglets (Liu et al., 2018), consistent with our results. The small intestine is the main location for digestion and absorption and is the most important absorption organ. The immature intestinal tract of weaned piglets is more susceptible to irritation, causing damage to intestinal morphology and barrier function (Hu et al., 2013). Impairing the intestinal morphology of piglets is mainly reflected by villus atrophy and increased crypt depth. VH was positively correlated with fasting weight gain and dry matter intake (Pluske et al., 1997), and a decrease in VH:CD is believed to be detrimental to nutrient digestion and absorption (Pluske et al., 1997; Montagne et al., 2003). Previous studies have shown that low doses of XOS (100 g/t) increase the villi height of weaned piglets (Su et al., 2021). In a previous study, feeding XOS (100 mg/kg) tended to reduce the ileum villi surface area, but the difference was not significant (Yin et al., 2019). Interestingly, our study showed a tendency for XOS to increase ileum VH, which may account for the increased ATTD of DM and GE.

The intestinal physical barrier is a complete epithelial structure (Vicente et al., 2001), composed of columnar epithelial cells such as absorption cells, goblet cells, and endocrine cells, as well as tight junctions (TJ) between cells (including *Claudins, occludin*, and *ZO-1*). *Claudins* form the structural skeleton of TJs, *occludin* is the main transmembrane protein found in TJs, and *ZO-1* is the basic scaffold protein of TJs (Suzuki, 2013; Turner et al., 2014). When TJs are damaged, intestinal permeability is increased, leading to intestinal dysfunction and host inflammation. Previous studies have found that XOS supplementation increased the mRNA expression levels of *claudin-3* and *occludin* in the ileum of broilers, and the mRNA expression levels of *occludin* and *ZO-1* in piglets (Chen et al., 2021b; Luo et al., 2021), suggesting that XOS enhances intestinal barrier function. Our study found that XOS can upregulate mRNA expression levels of TJ, indicating that XOS can block the passage of harmful substances such as antigens through intestinal mucosa.

As metabolites of intestinal flora, SCFAs play antibacterial, anti-tumor, and immune regulatory roles, and they are important for maintaining intestinal health. Previous studies have shown that XOS can significantly increase the levels of SCFAs in the large intestine of weaned piglets. For example, Wang et al. (2021) found that dietary supplementation of XOS (35%, 200 mg/kg) significantly increased the concentrations of AA, PA, BA, and VA in the cecum of weaned piglets. Similarly, we discovered that weaned piglets fed diets supplemented with XOS showed an increased concentration of AA and BA in the cecal contents and BA and PA in the colonic contents. XOS is reported to enhance the intestinal barrier and regulate intestinal immunity by activating host G protein-coupled receptor 109a or inhibiting histone deacetylase via microbiota-derived metabolites (including BA and PA) (Tang S. et al., 2022). SCFAs, especially BA, can reduce the risk of diarrhea and intestinal dehydration by stimulating the absorption of sodium and water in the gut (Pandey et al., 2015). SCFAs can promote the proliferation of colon cells and stimulate the proliferation and growth of small intestine cells (Sakata and Inagaki, 2001; Van den Abbeele et al., 2011). Moreover, they can improve the immune function by reducing oxygen levels (Makki et al., 2018). In the present study, weaned piglets fed XOS showed improved diarrhea and protected intestinal barrier, which may be closely related to the effects of SCFAs.

The microbiome is also part of the intestinal barrier, regulating the interactions between bacteria and intestinal cells and the absorption of nutrients (Scaldaferri et al., 2012). Prebiotics are defined as substances that selectively promote the growth of beneficial microorganisms, especially certain beneficial microorganisms (Laurell and Sjoberg, 2017). XOS is a highly effective prebiotic with health benefits and few side effects that exerts a positive influence on hindgut microbes. Li Z. et al. (2021) demonstrated that XOS could alleviate the negative effects of colitis by changing the posterior intestinal microflora *in vitro*. In our study, XOS enhanced OTUs and the alpha-diversity of intestinal microbes of weaned piglets. Diversity is generally considered necessary for stable ecosystems (McCann, 2000), as well as for microbial flora stability. Greater microbial diversity in the gastrointestinal tract may provide greater resilience and stability of the intestinal ecosystem, and the higher the microbial diversity in the gut, the greater the plasticity of immune responses (Bäckhed et al., 2005). Therefore, an XOS-induced increase in microbial diversity may play a positive role in gut health. In addition, PCoA showed significant clustering of XOS-treated samples, which further indicates that XOS has a profound effect on gut microbes.

To explore differences in microbial species between treatments, enriched bacteria in different treatment groups were analyzed by LEfSe. The number of disease-causing bacteria in colonic chyme was increased in the HP-treated group, including *p_Campylobacterota, o_Campylobacterales, c_Campylobacteria, f_Campylobacteraceae, g_Campylobacter*, and *g_Arthrobacter*. *Campylobacter* members are intestinal pathogens, and they are among the most common causative agents of foodborne gastroenteritis worldwide (Kaakoush et al., 2015). These bacteria adhere to oligosaccharide receptor sites in the intestinal tract, causing gastroenteritis via the erosion and/or secretion of toxins (Gibson et al., 2005). According to the correlation analysis results, the higher the diarrhea index, the stronger the Campylobacter abundance, which could be used to explain the high diarrhea index of piglets in the HP group and also elucidate the pathway of XOS that alleviates diarrhea. In addition, *g_Arthrobacter* members belong to the phylum Actinobacteria, whose presence indicates that piglets are prone to diarrhea (Karasova et al., 2021). The abundance of pathogenic bacteria *f_Peptostreptococcaceae* and *g_Clostridium_sensu_stricto_1* was increased in colonic contents in the LP treatment group. It is well documented that *f_Peptostreptococcaceae* and *g_Clostridium_sensu_stricto_1* are enriched in the intestinal cavity of patients with colon cancer compared with normal people (Chen et al., 2012). The relative abundance of *Proteobacteria*, elevated during intestinal disease, was decreased in the colonic contents in piglets fed XOS-supplemented diets compared with those fed diets without XOS (Rizzatti et al., 2017). Previous studies demonstrated that 100 g/t XOS supplementation during the growing and fattening periods of pigs significantly reduced the relative abundances of presumably pathogenic bacteria (*Proteobacteria*) (Pan et al., 2019). Some researchers have suggested that an increased abundance of *Proteobacteria* is a sign of microbial imbalance and a potential diagnostic criterion for diarrhea (Shin et al., 2015). In conclusion, feeding the HP diet significantly increased the diarrhea index of piglets, which may be related to intestinal barrier damage caused by the accumulation of pathogenic bacteria. However, XOS supplementation in the HP diet can greatly reduce the accumulation of pathogenic bacteria, thus stabilizing the gut ecosystem.

## 5. Conclusion

In this study, we tested different levels of plant-based proteins and XOS addition in the diets of piglets and found that high levels of plant-based protein can aggravate diarrhea in weaned piglets, and the addition of XOS had no significant effect on the growth performance of weaned piglets, but it ameliorated the adverse effects of plant protein on piglets by increasing nutrient digestibility, intestinal barrier function, levels of SCFAs in the large intestine, and reducing the abundance of harmful bacteria.

## Data availability statement

The data presented in the study are deposited in the National Center for Biotechnology Information (NCBI) Sequence Read Archive (SRA), accession number: PRJNA967666.

## Ethics statement

The animal study was reviewed and approved by Sichuan Agricultural University Institutional Animal Care and Use Committee. Written informed consent was obtained from the owners for the participation of their animals in this study.

## Author contributions

DW, QW, and YL conceived the project and designed the experiments. QW, YZha, and LG carried out animal experiments and performed laboratory work. QW, XM, YY, YZhu, XJ, LH, JL, SX, BF, ZF, and LC performed the statistical analysis. QW wrote and DW revised the manuscript. All authors critically reviewed the manuscript and gave final approval for the version to be published.
