# Supplementary material for: Xylo-oligosaccharides improve the adverse effects of plant-based proteins on weaned piglet health by maintaining the intestinal barrier and inhibiting harmful bacterial growth
Source: Front Microbiol. 2023 May 25;14:1189434. doi: 10.3389/fmicb.2023.1189434 (PMC10249996; doi:10.3389/fmicb.2023.1189434)
Supplement: Supplementary file 1 [file Data_Sheet_1.docx]

**Supplementary TABLE S1** Primer sequences of the target and reference genes

| **Gene** | **Product length/bp** | **Accession number** | **Primer Pairs (5' to 3' direction)^1^** |
| --- | --- | --- | --- |
| *GAPDH* | 220 | NM_001206359.1 | F: CGTCCCTGAGACACGATGGT  R: CCCGATGCGGCCAAAT |
| *ZO-1* | 114 | AJ318101 | F: CCGCCTCCTGAGTTTGATAG  R: CAGCTTTAGGCACTGTGCTG |
| *Claudin-1* | 106 | NM_001244539.1 | F: GATCGGCTCCATCGTCAGCA  R: CATTGACTGGGGTCATGGGGTC |
| *Occludin* | 158 | NM_0011636471 | F: TTCATTGCTGCATTGGTGAT  R: ACCATCACACCCAGGATAGC |

^1^F , forward primer sequence (5′→ 3′); R, reverse primer sequence (5′→ 3′).


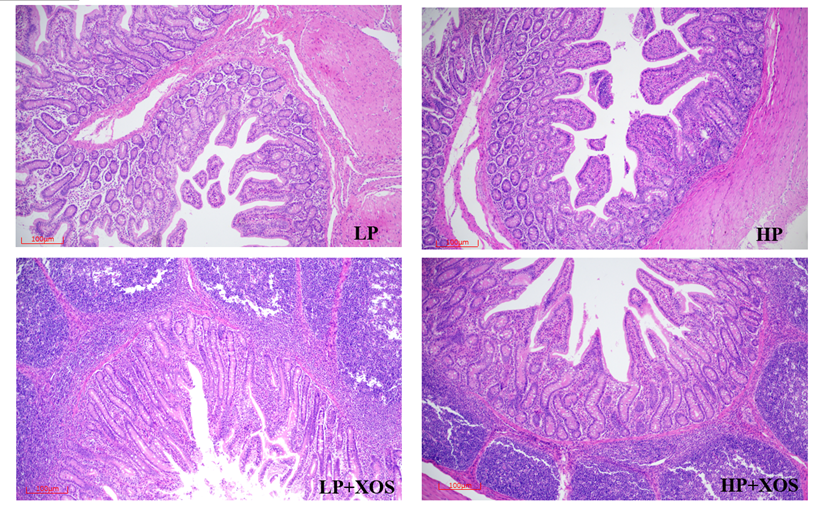


**Supplementary Figure S1** Effects of different levels of plant-based proteins diets supplemented with XOS on intestinal morphology in weaned piglets.
